# Categorical Smoothness of 4-Manifolds from Quantum Symmetries and the Information Loss Paradox

**DOI:** 10.3390/e24030391

**Published:** 2022-03-11

**Authors:** Jerzy Król, Torsten Asselmeyer-Maluga

**Affiliations:** 1 Chair of Cognitive Science and Mathematical Modeling, University of Information Technology and Management, ul. Sucharskiego 2, 35-225 Rzeszw, Poland; 2 German Aerospace Center (DLR), Rosa-Luxemburg-Str. 2, D-10178 Berlin, Germany; torsten.asselmeyer-maluga@dlr.de

**Keywords:** quantum spacetime structure, exotic 4-smoothness, Boolean ultrapowers, Basel topos, quantum mechanics, information loss

## Abstract

In this paper, we focus on some aspects of the relation of spacetime and quantum mechanics and the study counterparts (in Set) of the categorical local symmetries of smooth 4-manifolds. In the set-theoretic limit, there emerge some exotic smoothness structures on $R^{4}$ (hence the Riemannian nonvanishing curvature), which fit well with the quantum mechanical lattice of projections on infinite-dimensional Hilbert spaces. The method we follow is formalization localized on the open covers of the spacetime manifold. We discuss our findings in the context of the information paradox assigned to evaporating black holes. A black hole can evaporate entirely, but the smoothness structure of spacetime will be altered and, in this way, the missing information about the initial states of matter forming the black hole will be encoded. Thus, the possible global geometric remnant of black holes in spacetime is recognized as exotic 4-smoothness. The full-fledged verification of this proposal will presumably be possible within the scope of future quantum gravity theory research.

## 1. Introduction

The proper description of the regime whereby classical spacetime (a smooth Lorentzian 4-manifold) itself overlaps with the quantum regime of reality and becomes subject to quantum rules is currently one of the biggest unsolved puzzles in theoretical physics. A partial understanding of this can be obtained within the framework of existing attempts to describe quantum gravity (QG), such as superstring theory or loop QG; however, so far we do not have access to sufficient observational data to discriminate between these approaches or direct future attempts to create a proper QG theory. This is why certain, sometimes subtle, indications may come from the mathematics of very successful physical theories, such as quantum field theory or quantum mechanics (QM), which are embedded in the field of operator algebras, and general relativity (GR), which is from the field of differential geometry and topology. One such indication is the fact that dimension 4 is quite unusual in mathematics and there exist nondiffeomorphic copies of smooth $R^{4}$s (exotic $R^{4}$s), while there are no such exotics for any other $R^{n},n\neq 4$. There is certainly no a priori need to use exotic smoothness in physics; however, such an approach has been used successfully for several years now (since the 1980s, when $R^{4}$s were found to exist by mathematicians)—e.g., [1,2,3,4]. What focuses our attention on $R^{4}$s is that spacetime allowing for exotic smoothness in dimension 4 significantly breaks the distinction between classical and quantum regimes, and this phenomenon paves the path for an alternative approach to QG called *smooth QG* [5]. In the present work, we deal with yet another clue which mathematics can give. It is always possible to try to understand the discrepancies emerging between physical theories such as QM and GR with increasing degrees of formalization. As the result, formal aspects, such as those connected with the models of theories, might appear that do not match between the theories and consequently the formal possible resolutions of mathematics yields. Again, there is no ’a priori’ necessity to make use of highly formal techniques, although, in spite of the lack of sufficiently good alternatives or, rather, when various alternatives fail in the search for QG, such possibilities should be followed. This has already led to promising results—e.g., [6,7].

In this paper, we focus on the formalization of spacetime as well as the formalization of QM, but we also employ the category theory point of view on the smoothness of 4-manifolds proposed in [8] as a starting point. Formalizations follow the rules of axiomatic Zermelo–Fraenkel set theory (ZFC-C for the axiom of choice), especially the models thereof, which appear as valid carriers of the underlying physics. In this respect, the present work is the continuation of certain previous results [6,7,9,10,11]. The perspective chosen allows for the uniform treatment of spacetime both as a classical entity and as part of the QM regime. This approach can be applied for understanding the information loss paradox for black holes in an entirely new way, which is discussed here briefly, but a thorough analysis will be provided elsewhere.

**Figure 1:**
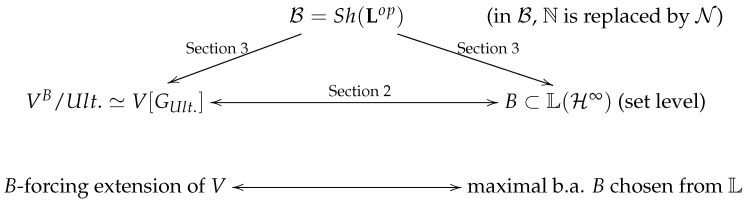
For the infinite-dim. Hilbert space, the lattice $L(H^{\infty })$ contains maximal b.a. *B*s which support non-trivial *B*-forcings.In the Basel topos B, the effective NNO is the smooth object N, which is an (intuitionistic) nonstandard extension of N.

Figure 1 shows the logical structure of this paper. The data represented by the graph are localized on a smooth manifold (a region of spacetime). Thus, typically one understands that for any open good cover $U_{\alpha },\alpha \in I$ of the region (such as $R^{4}$), there are such indices α≠β where the Basel topos B is assigned to α and the topos Set to β. Thus, the upper node $B=Sh(L^{op})$ represents the topos which $U_{\alpha }$ is assigned to internally. This is briefly recalled in the next section, where we describe in accordance with [8] the construction of categorical local external/internal smooth manifolds and their relation to exotic 4-smoothness on open 4-manifolds. Basically, the relation of the lattice of projections L on the infinite-dimensional H and the forcing extensions of ZFC models *M* of local data has been analyzed in some papers (e.g., [6,10,12]). This is why we report on this in the key terminologies section below, which aims to provide a brief overview of the state of the art in this subject and the methods applied. The factorization of this through the topos B, still localized on a spacetime manifold, is discussed in the Results Section 3. One of the new formal aspects of this approach is the well-foundedness of the models and their real numbers objects. Well-foundedness is, in fact, a gluing property of the externalizations of real numbers emerging from the topos and their forcing extensions in the Set. The main result is stated in Theorem 1, where it is shown that the smoothness resulting from the QM lattice L is in fact the locally B modified 4-smoothness of spacetime. Finally, armed with this result and the factorization above, we can understand the relation of the smooth spacetime manifold in the quantum regime and the large exotic $R^{4}$s (not necessary small), which allows us to provide an alternative perspective of the information loss paradox of black holes (BH) in Section 4. Thus, we have found a distinguished smoothness structure which applies to large scales as well to the quantum regime and which cannot be the standard $R^{4}$ (Theorem 1). This structure carries information about quantum matter fields and their energy–momentum content across 4-spacetime regions. In the Appendix A, we illustrate the phenomenon in the case of Schwarzschild BH.

## 2. Categorical Smoothness on Open 4-Manifolds: Key Terminologies

In this section, we group together some facts regarding the relation of exotic smoothness structures on open 4-manifolds and the local categorical modifications of the manifolds by the smooth topos B. The topos B and its close variants have been analyzed extensively in [13], where the authors considered the modification of the object of natural numbers (NNO), and replacing N with N. This opened up the possibility of dealing directly with smooth functions in exotic structures [8]. This modification of the standard N (though in the intuitionistic framework) is an exceptional feature of B compared to the other toposes considered in the book. The topos perspective on exotic 4-smoothness has been analyzed in our previous publication [8].

**Remark** **1.** *Exotic $R^{4}$ is a smooth Riemannian 4-manifold where no diffeomorphism $\phi :R^{4}\to R^{4}$ exists from it onto the standard smooth 4-space, even though $R^{4}$ and $R^{4}$ are homeomorphic. There are two classes of them: small $R^{4}$s (a continuum of many of them), which are embeddable into $R^{4}$ (or equivalently $S^{4}$), and large $R^{4}$s (structured continuous families of many of them), which do not allow for such embeddings. Small $R^{4}$s are typically understood via handle decompositions and it is widely recognized that they contain some infinite towers of 2-handles, which are called Casson handles. Large $R^{4}$s are far less well understood [14].*

One reason for developing an alternative to the hendlebody decomposition procedures of generating exotic $R^{4}$s is the fact that if exotic $S^{4}$ exists and we remove one point from it we are left with exotic $R^{4}$, which does not follow anything we know about exotic $R^{4}$s from the usual handle decompositions.

**Remark** **2.** *One unsolved aspect of one of the millennium problems of the Clay Institute is whether exotic 4-sphere $S^{4}$ exist. If they do, which generally expected, another kind of exotic $R^{4}$s completely different to the large or small ones should also exist [14]. In particular, the 1-point compactification of any large or small $R^{4}$ (i.e., $S^{4}$) is always smooth $S^{4}$.*

We allow for the local modification of smoothness structures, especially on $R^{4}$, by toposes such as B. This is a smooth topos and it ’contains’, in a categorical sense, all smoothness structures on any $R^{n},n=1,2,3,4,\ldots$. The procedure is presented and analyzed in [8]. Let us recall it briefly. A topological *n*-dimensional manifold is a Hausdorff, metrizable topological space *M* together with an atlas of charts ${\left\{\left(U_{\alpha },\phi_{\alpha }\right)\right\}}_{\alpha \in I}$, where $M={⋃}_{\alpha \in I}U_{\alpha }$ and the maps $\phi_{\alpha }:U_{\alpha }\to \phi \left(U_{\alpha }\right)\subseteq R^{n}$ are homeomorphisms for open subsets of $R^{n}$.

An atlas is smooth if, for all α,β∈I and $U_{\alpha \beta }=U_{\alpha }\cap U_{\beta }\neq \emptyset$, the maps

(1) $$\phi_{\alpha \beta }:=\phi_{\beta }\circ \phi_{\alpha }^{-1}{|}_{\phi_{\alpha }\left(U_{\alpha \beta }\right)}:\phi_{\alpha }\left(U_{\alpha \beta }\right)\to \phi_{\beta }\left(U_{\alpha \beta }\right)\;.$$

are smooth and ${\left(\phi_{\alpha \beta }\right)}^{-1}=\phi_{\beta \alpha }$ are smooth as well.

The maximal atlas ${\left\{\left(U_{\alpha },\phi_{\alpha }\right)\right\}}_{\alpha \in I}$ of local smooth charts is called the smooth structure of *M*.

To define a category, one needs the class of objects and morphisms (arrows) fulfilling certain natural properties [13]. Set will be the category of sets and functions and M will be the category of smooth manifolds and smooth maps [13]. Special categories, resembling Set, are toposes. Set is a topos by itself and B is a Grothendieck topos—i.e., the category of sheaves on a site where site is a base category. For B, it is the category of loci—i.e., the opposite category to the category of smooth rings and smooth morphisms between them. For more details, the reader can review various textbooks such as [13].

There exists an embedding, *s*, of the category M into the topos B, such that (see [8,13])

$$M∋M\to s\left(M\right)=\underline{M}:=B\left(-,\hat{s}\left(M\right)\right)\;\mathrm{in}\;B$$

where $B(-,\hat{s}\left(M\right))$ is the set of all morphisms in B ending at $\hat{s}\left(M\right)$ the loci in L, which is due to the smooth ring representing *M*.

The embedding s:M↪B is full and faithful [13] (Lemma 5.2, p. 286).

Let M∈M be a smooth manifold with a smooth atlas ${\left\{\left(U_{\alpha },\phi_{\alpha }\right)\right\}}_{\alpha \in I}$ (in Set). Following [8], we assign to any α∈I a map s:M→B, $s:U_{\alpha }\to s\left(U_{\alpha }\right)$ or the identity (isomorhism in Set, diffeomorphism) $i:U_{\alpha }\to U_{\alpha }$ in Set. Thus, the following function is defined:

(2) $$\forall \left(\alpha \in I\right)U_{\alpha }\mapsto \left\{\begin{array}{cc} i\left(U_{\alpha }\right)≃U_{\alpha }, & \mathrm{for}\;\alpha \in A\subset I\\ s(U_{\alpha })\in B, & \mathrm{for}\;\alpha \in I\backslash A\;. \end{array}\right.$$

Then, we define (following [8]) the B-cover of a smooth manifold *M* as the above assignment (2), such that A≠∅ and A≠I—i.e.,

$$\exists \left(\alpha ,\beta \in I\wedge \alpha \neq \beta \right)U_{\alpha }\mapsto U_{\alpha }\;\mathrm{and}\;U_{\beta }\mapsto s\left(U_{\beta }\right)\;.$$

Then, the B-local smooth manifold is a smooth manifold $\hat{M}$ which every atlas has underlying B-cover—i.e.,

∀(UanopengoodcoverofM)∃(B-coverdefinedfromU).

At first, it might seem that $\hat{M}$ and *M* are equivalent (diffeomorphic) manifolds in Set. The direct though important observation of [8] gives the following result:

**Lemma** **1.** *Let $M=R^{n}$. Then, $\hat{M}$ is not diffeomorphic to M.*

The conclusion is that $\hat{M}$ has to be an exotic $R^{4}$. Moreover, when the modification of $R^{4}$ by B is global, the resulting smooth manifold can be the standard $R^{4}$. Thus, the local B property vs. global is the true obstruction for choosing the standard smoothness on $R^{4}$.

One of the important guiding principles in relating 4-smoothness structures and smooth toposes is the modification of the NNO N→N to the smooth object of natural numbers N, which in Set corresponds to the ultrafilter construction. More precisely, there is the canonical object of real numbers in B, $R_{B}$, from which there follows the extension of N [13]. Since, internally to B, one cannot make any use of the axiom of choice (AC), the ultrafilter construction (ultraproduct) is not fully constructivistic; however, externally to B in Set, we have the counterparts of N and $R_{B}$, which are nonstandard extensions ${}^{*}N$ and ${}^{*}R$ given as the ultraproducts. Thus, one can preserve the nonstandardness of number objects when leaving B. By applying usual global functor construction Γ:B→Set, one always obtains standard N and R. Here, there follows the Boolean ultrapower constructions which generalize the usual Boolean-valued models in, for example, ZFC, $V^{B}$, as well as the ultrapowers usually used to build nonstandard models such as ${}^{*}N$ and ${}^{*}R$.

Boolean-valued models of ZFC, $V^{B}$, were invented as suitable tools to describe forcing extensions (Scott, Solovay, Vop$\overset{˘}{e}$nka; see [15,16,17]) of a ZFC model *V*. Here, *B* is a complete Boolean algebra. It has been observed that, given a filter *U* in *B* that is *V*-generic, the quotient model $V^{B}/U$ is the 2-valued forcing extension isomorphic to M[U] (here, *U* can represent some generic reals as in, e.g., Cohen or random forcings). There is, however, a subtlety here—namely, in the case of *V*, which is the universe of all sets, it is impossible to add more sets. The solution is well recognized and one defines a ’smaller’ universe $\overset{ˇ}{V}$ in *V*, such that now the Boolean ultraproduct describes the proper extension $\overset{ˇ}{V}\left[U\right]$ and *U* is $\overset{ˇ}{V}$-generic and $U\notin \overset{ˇ}{V}$ (e.g., [15,17,18]). Given any complete Boolean algebra and a universe of sets *V* (such as the cumulative hierarchy *V* of all sets, or a transitive model *M* of ZFC), there always exists a Boolean-valued model $V^{B}$ of ZFC. $V^{B}$ comprises the class of Boolean names τ, such that τ is the set of pairs 〈t,b〉, where *t* is a *B*-name and b∈B and this recursion defines the entire class. Then, one assigns the Boolean values 〚ϕ〛 to formulas ϕ of ZFC starting from atomic ones [15] and using the completeness of *B* with ⋁ (⋀) the supremum (infimum) in *B*:

(3) $$\begin{array}{c} 〚\sigma \in \tau 〛=\underset{\langle e,b\rangle \in \tau }{⋁}〚\sigma =e〛\wedge b\\ 〚\sigma =\tau 〛=〚\sigma \subseteq \tau 〛\wedge 〚\tau \subseteq \sigma 〛\\ 〚\sigma \subseteq \tau 〛=\underset{e\in \mathrm{dom}(\sigma )}{⋀}\left(〚e\in \sigma 〛\to 〚e\in \tau 〛\right). \end{array}$$

Then, every axiom of ZFC has a Boolean value of 1 in $V^{B}$ for any complete Boolean algebra *B* [15] (Theorem 3).

One way of embedding *V* into $V^{B}$ is by taking check names of sets in *V* recursively and defining $\overset{ˇ}{V}$ the universe of check names as

$$V∋x\mapsto \overset{ˇ}{x}=\left\{\langle \overset{ˇ}{y},1\rangle :y\in x\right\}\in \overset{ˇ}{V}$$

The Boolean value in $V^{B}$ of $\tau \in \overset{ˇ}{V}$ is calculated as $〚\tau \in \overset{ˇ}{V}〛={⋁}_{x\in V}〚\tau =\overset{ˇ}{x}〛$, so that one proves in $V^{B}$ [15] (Lemma 6)

$$〚\overset{ˇ}{V}\;\mathrm{is}\;a\;\mathrm{transitive}\;\mathrm{class}\;\mathrm{containing}\;\mathrm{all}\;\mathrm{ordinals}〛=1\;.$$

The other way to embed $V\to V^{B}$ is by $\left\{\overset{ˇ}{x}:x\in V\right\}$, which is 2-valued model $V^{2}$ where 2={0,1} is the 2-value Boolean algebra which is the complete subalgebra of any *B*.

Given an ultrafilter *U* in *B*, we create the congruence relations ${=}_{U}$ and $\in_{U}$ in $V^{B}$ as:

$$\sigma {=}_{U}\tau :=〚\sigma =\tau 〛\in U;\;\;\;\;\sigma \in_{U}\tau :=〚\sigma \in \tau 〛\in U.$$

For any τ∈V, we define ${\left[\tau \right]}_{U}$ the restricted equivalence class composed of all this σ∈V, such that σ has the minimal possible rank in the cumulative hierarchy of sets and 〚σ=τ〛∈U. Then, for any ultrafilter *U* on *B*, the universe ${\overset{ˇ}{V}}_{U}$ is defined as:

$${\overset{ˇ}{V}}_{U}:=\left\{{\left[\tau \right]}_{U}:〚\tau \in \overset{ˇ}{V}〛\in U\right\}.$$

Note that the map $j_{U}:V\to {\overset{ˇ}{V}}_{U}$ is given by $V∋x\mapsto {\left[\overset{ˇ}{x}\right]}_{U}\in {\overset{ˇ}{V}}_{U}$ and that $\in_{U}$ is the proper (not necessary standard) relation in ${\overset{ˇ}{V}}_{U}$. Then, the *Boolean ultrapower of V by the ultrafilter U on B* is the pair $\left({\overset{ˇ}{V}}_{U},j_{U}\right)$.

**Remark** **3.** *${\overset{ˇ}{V}}_{U}$ is not necessarily equal to $\left\{\left[\overset{ˇ}{x}\right]:x\in V\right\}$; $V^{B}/U$ is the forcing extension of ${\overset{ˇ}{V}}_{U}$, $V^{B}/U={\overset{ˇ}{V}}_{U}\left[G\right]$ [15].*

The most important feature of the entire construction is that Boolean ultrapowers can be equivalently described purely algebraically, without taking care of the *V*-genericity of *U* or the internal completeness of the Boolean algebra *B*. This is precisely the point of view which follows the ultraproduct construction in model theory [19]. Both approaches—generic, where forcing is described by $V^{B}/U$, with *U* being $\overset{ˇ}{V}$-generic, and the ultraproduct with respect to an arbitrary filter in *B* (which in particular gives rise to ${}^{*}N$, ${}^{*}R$)—are equivalent, as shown in [15] (Theorem 30).

## 3. Results

Before stating our main results, we need to turn to the quantum regime of spacetime smooth manifold $M^{4}$. This requires some explanation. We consider QM on Hilbert spaces with special emphasis on the case of infinite-dimensional H. This may seem to contradict the fact of black holes regimes emerging in spacetime, but the approach described here is based on the spacetime which still bears features of it even in the quantum regime. This is why we consider the system composed of BHs and spacetime where Heisenberg uncertainty can still apply. Thus, infinite-dimensional H is a natural choice for such a stage of spacetime. We do not decide here whether there is a primordial stage of BHs entirely assigned to finite-dimensional Hilbert spaces of states without spacetime degrees of freedom and whether spacetime somehow eventually emerges from it. However, such questions can also be analyzed based on the formal tools described in this work. Thus, as a rule we assign dimH=∞ in the analysis of spacetime in the quantum regime. There are formal results concerning it which are important here (e.g., [6,9,10,12]). We perform formalization by applying ZFC and models of ZFC; however, this leads to the *forcing* degrees of freedom, since there are now two possible perspectives [6]. The internal perspective is the formal-language and certain (natural) model of ZF(C) in which we formalize, while the external perspective is where there is a set of models (derived from the local formalizations in spacetime and in the lattice) and the relations between the models. This external perspective refers directly to *forcing* in set theory, since models are changed by forcing.

The analysis of QM based on the lattice of projections L (uniquely determined by the Hilbert space of states) was performed and the formalization procedure was set as the canonical means of deriving ZFC models from L and showing that they support nontrivial forcing. We refer interested readers to [6,10] for an extended discussion of the formalization and forcing in QM and to textbooks such as [17,20] regarding forcing in set theory.

**Remark** **4.** *(L,∧,∨,¬,0,1) is a nondistributive lattice for dimH≥2 comprising projections p:H→H on the closed linear subspaces, p∘p=p. The infimum ∧ is the set operation ∩ on subspaces, the supremum ∨ is the span of the set sums of subspaces, ¬p=1−p, where 1 is the identity on H and 0 is the projection on ∅. One can always choose maximal complete Boolean algebras (blocks) from L, such that every p∈L belongs to some block $B_{p}\subset L$.*

Now, we consider the family of blocks $\left\{B_{k},k\in K\right\}$ covering the entire lattice. Thus, $\forall_{p\in L}\exists_{k\in K}p\in B_{k}$. This is a family of classical local contexts in QM, since for every family of commuting self-adjoint operators $A_{\alpha },\alpha \in I$, there always exists a maximal, complete Boolean algebra of projections (a block), such that each $A_{\alpha }$ in the family is represented by the spectral decomposition $A_{\alpha }=\int \lambda dE_{\lambda }^{\alpha }$, where each spectral measure $E^{\alpha }$ takes values in projections from $B_{k},k\in K$ (i.e., $dE_{\lambda }^{\alpha }\in B_{k}$). Thus, we obtain any self-adjoint (s.a.) operator on H making use of blocks—i.e., to every s.a. operator *A*, there exists a block determining *A*. This covering property of blocks for L is the counterpart to the local patches covering the smooth spacetime $M^{4}$.

Of special importance is the more precise understanding of blocks, since they carry valid information regarding forcing and models of ZFC. Let *B* be the algebra of Borel subsets of [0,1] modulo the ideal $N_{0}$ of μ-measure zero Borel subsets (the measure algebra)

(4) $$B=Bor\left(\left[0,1\right]\right){/}_{N_{0}}\;.$$

**Lemma** **2** ([21,22]). *B is an atomless Boolean algebra.*

There are two separated cases regarding the atomicity of blocks, corresponding to finite and infinite dimensions of H:

(1) If dimH=∞, the blocks in L have the general form
  $$B=B_{a}⊕B,$$
  where $B_{a}$ is an atomic Boolean algebra of 1-dim. projections and $B=Bor\left(\left[0,1\right]\right){/}_{N_{0}}$.
(2) If dimH<∞, then the blocks in L are always fully atomic [21,22].

The measure algebra *B* distinguishes the infinite dimension of H. However, the atomless property of *B* is also responsible for set theory forcing coming into the picture. Why is this so? The ZFC models help us to grasp degrees of incompleteness of information contained in local contexts—i.e., blocks. Namely, where there had existed a homomorphism $h_{L}:L\to \left\{0,1\right\}$ of the entire lattice to the 2-element Boolean algebra, then by truncation to the contexts there would exist a family of $h_{\alpha }:B_{\alpha }\to \left\{0,1\right\}$ which would agree between the contexts giving rise to $h_{L}$. Thus, this would lead to the existence of a dispersion-free state, which, in particular, would reduce QM to a classical theory (there would exist local hidden variables for QM). We (following [6]) formalize local contexts $\left\{B_{\alpha }\right\}$ and their homomorphisms $\left\{h_{\alpha }\right\}$ in transitive standard models $\left\{M_{\alpha }\right\}$ prior to the question about their global agreement. It follows that [6]:

1. For $B_{\alpha }$ in $M_{\alpha }$ and $h_{\alpha }$ to be completely additive, $h_{\alpha }^{-1}\left(1\right)$ cannot be in $M_{\alpha }$ (no transitive standard model containing $h_{\alpha }$ and $B_{\alpha }$ exists). Thus, $h_{\alpha }$ is not in $M_{\alpha }$ either.
2. $h_{\alpha }^{-1}\left(1\right)=G_{\alpha }$ is a generic ultrafilter in $B_{\alpha }$, meaning that $h_{\alpha }\in M_{\alpha }\left[G_{i}\right]$—the forcing extension of $M_{\alpha }$ with respect to $B_{\alpha }$ (but $h_{\alpha }\notin M_{\alpha }$).
3. Since $B_{\alpha }=B$, $M_{\alpha }\left[G_{i}\right]$ is the random forcing extension of $M_{\alpha }$.
4. For dimH=∞, there has to exist a family $\left\{M_{\alpha }\left[G_{i}\right]\right\}$ of random forcing extensions locally describing the lattice L(H). This family can not be a single-element family. In fact, there are many such forcing extensions of $M_{\alpha }$ for i=1,2,3,….
5. Finally, one considers the equivalent formulation of forcing in terms of the Boolean valued models of ZFC, $V^{B}$ (e.g., [17,18]). This also works in the universe (standard, transitive) of sets such as *V* and its random extensions $V[G_{i}]$.

Thus the following two regimes were assigned to the smooth manifold’s spacetime. One semi-classical (non-quantum, intuitionistic) regime is represented by locally modified smooth open 4-manifolds such as $R^{4}$, as discussed in the previous section, and the other quantum and 2-valued regimes derived from the quantum mechanical lattice of projections. This last applies for the infinite-dimensional Hilbert space H and is represented by the collection of local generalized coordinate frames described by the collection of ZFC models, which are forcing extensions of *V*. Each model $V[G_{i}]$ hosts $R_{i}^{4},i=1,2,3,\ldots$. The semi-classical spacetime is described by the collection of local frames—i.e., $R_{B,j}^{4};j=1,2,3,\ldots$.

In the remaining part of this section, we will connect the two pictures by finding the way from B to Set, which, however, does not follow the usual global section functor [13]. As a result, there follows a precisely 2-valued limit of semi-classical (B-local) spacetime which appears to agree with the described above local forcing extensions derived from the quantum limit. This is a remarkable result, indicating that to fully understand discrepancy of quantum and classical regimes of spacetime, we need to turn to

1. unique dimension 4 for spacetime;
2. smoothness structures of spacetime;
3. formal tools of set theory applied to QM;
4. formal tools of set theory applied to 4-dim. smoothness structures.

To realize the above, we will show (the 2-valued limit of the B-local manifold *M* is called B-invariant set-based manifold *M*):

**Theorem** **1.** *A smoothness structure on $R^{4}$, which agrees with QM on infinite-dimensional Hilbert spaces, shows some B-invariant set-based smoothing of $R^{4}$.*

In Figure 1, we present the main line of argumentation we follow here (the solid line). The arrows connect the LHS R (well-founded, 2-valued) with the RHS $R_{V[Ult]}$, which is also well-founded and 2-valued but is the object of real numbers in the random forcing extension model V[Ult]. The left side of the figure corresponds to the classical GR regime (with the local B-modifications of spacetime more toward the centre). The left side corresponds to the random forcing extensions of *V* derived from the lattice L; thus, the line connects both regimes, classical and quantum. Note that the other possible paths from R on LHS end with the standard $R^{4}$ on the RHS, which, however, do not match the forcing extension, hence QM.

The proof of Theorem 1 comprises a few lemmas below, some of which are quite direct presentations of known results about the topos B, nonstandard natural and real numbers, or direct quotations of the results obtained for Boolean ultraproducts; however, together they help us to grasp the general idea behind the Theorem 1.

Let ${}^{*}N$ be a nonstandard (in a sense of A. Robinson infinitesimal analysis) set of natural numbers. This set in general is an end extension of the set N of standard natural numbers and can be obtained as the ultraproduct construction $N^{\omega }/Ult$, where Ult is a nonprincipal ultrafilter on N (i.e., on P(N)). Similarly, ${}^{*}R$ is construed as $R^{\omega }/Ult$. ${}^{*}N$ and ${}^{*}R$ are both non-wellfounded, though they share all the first-order properties with their N,R counterparts. Internally to B, the NNO becomes smooth N and R becomes the smooth R. In fact, the canonical object in B is R, which is a representable smooth ring $C^{\infty }\left(R\right)$ in L.

**Lemma** **3.** *The smooth N in B is nostandard—i.e., N contains (in a categorical sens) infinitely large natural numbers. The smooth R in B is a nonstandard set of real numbers—i.e., it contains infinitely large real numbers and its inverses infinitesimally small real numbers (invertible infinitesimals) in B.*

The proof of this lemma relies on the forcing of the existence of invertible nonstandard numbers in B and can be found in [13] (pp. 285–286) (see Remarks 5 and 6 below).

**Remark** **5.** *R in B contains also noninvertible infinitesimals: nilpotent ones (in the sense of A. Kock). In fact, the coexistence of both kind of infinitesimals was one of the reasons behind developing the smooth infinitesimal analysis by Moerdijk and Reyes [13].*

In the absence of AC in B, we still describe the object of smooth real numbers as the quotient smooth ring in the site L. The site is then embedded in Sh(B)=B via Yoneda embedding.

**Remark** **6.** *The site B for the topos B is, in fact, the category of loci L, where a special Grothendieck topology allowing for the existence of invertible infinitesimals in B was chosen. Thus, there are nonstandard reals and naturals. This is because the intuitionistic logic allows in general for non-inhabited objects. The topology of B forces the existence of the infinitesimals in B [13] (pp. 285–286)*

We say that two structures, *A* and *B*, are elementarily equivalent, A≡B, if all the first-order sentences valid in *A* are also valid in *B* and vice versa.

**Lemma** **4.** ${}^{*}R$ is elementarily equivalent to R, ${}^{*}R\equiv R$; however, ${}^{*}R$ is a non-well founded set of real numbers for certain non-principal ultrafilter Ult on P(N).

The proof of this lemma follows directly from Łoś theorem with regard to the transfer principle and the basic properties of ultraproducts [23].

**Figure 2:**
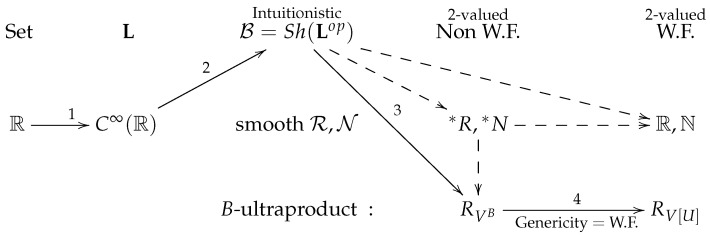
The solid arrows follow the way we argue in proving Theorem 1. This shows that the smoothness of $R^{4}$ agreeing with quantum lattice L (forcing extensions) is given by the well-founded (W.F.) 2-valued limit of local B-modifications of $R^{4}$.

In the next lemma, we deal with Boolean ultrapowers [15,18,19] which generalize both the ordinary ultrapowers of structures and the Boolean-valued models of set theory (see also Section 2 for definitions). Since forcing is canonically formulated via Boolean-valued models and nonstandard extensions of N,R can come from ultraproduct constructions. Thus, the Boolean ultraproducts are well-adapted to the case considered here. Namely, given the local B-modifications of the smooth structure of spacetime and taking a 2-valued limit, we consider a stage where there are nonstandard ${}^{*}N$ and ${}^{*}R$. On the other hand, we have a quantum limit where there are forcing extensions of R (relative to models of ZFC). In general, given the nonstandard models of real numbers (2-valued), if these are generated via an ultrapower method, there exists a certain utrafilter Ult on P(N), such that ${}^{*}R≃R^{\omega }/Ult$. Whenever Ult is non-principal, the resulting ${}^{*}R$ is non-wellfounded. In the scope of Boolean ultrapowers, they also serve as a method of building models of ZFC, which can be nonwellfounded depending on an ultrafilter *U* on *B*; thus, we replace ultrafilters on P(N) with certain ultrafilters *U* on general Boolean algebras—here, atomless *B*. If the models are nonwellfounded, the corresponding ultrafilters *U* are called nonwellfounded. Finally we want to land in a wellfounded model of ZFC with a wellfounded set of reals. In Figure 2, solid arrow 3 represents the retrieving wellfounded 2-valued R from the internal to B smooth *R*. This goes through the intermediate stage of the nonwellfounded Boolean ultraproduct as model of ZFC (which is not clearly shown in Figure 2).

**Lemma** **5.** *The generic extension of R with respect to the ZFC Boolean model $V^{B}$ (in Set—i.e., reals R in $V^{B}/U$) is the wellfounded reduct of the non-wellfounded Boolean ultrapower $R_{\hat{U}}$ relative to a Boolean algebra B ($\hat{U}\subset B$ nonwellfounded).*

This is, in fact, the core of the entire construction and relies on the deep connection between the ultraproducts, wellfoundedness, Boolean ultraproducts, and forcing extensions. This follows from the three results of [15], which we present below.

**Lemma** **6** ([15], Theorem 27)**.** *Every infinite complete Boolean algebra B admits nonwellfounded ultrafilters.*

One clearly concludes that in $B=Bor\left(\left[0,1\right]\right)/N_{0}$, there has to exist nonwellfounded ultrafilter, and thus it can support (generate) nonstandard ${}^{*}N$ and ${}^{*}R$ when outside of B. Now, we are looking for the consistent environment for the standard N, R derived from the above Boolean ultrapower based on *B* and the nonwellfounded ultrafilter. To this end, it is enough to observe that requiring standardness of N suffices, since it holds true that

**Lemma** **7** ([15], Theorem 23)**.** *If U is an ultrafilter in V (the universe of sets) on the complete Boolean algebra B, then the following are equivalent:*

*1.*  *The Boolean ultrapower ${\overset{ˇ}{V}}_{U}$ is wellfounded.*
*2.*  *The Boolean ultrapower ${\overset{ˇ}{V}}_{U}$ is an ω-model—that is, it has only standard natural numbers.*

One concludes that, given standard natural numbers N in any $\overset{ˇ}{V}$, it has to be wellfounded and contain the standard R. This property excludes nonstandard R in any wellfounded model $\overset{ˇ}{V}$ externally to B, provided there is a standard N. Internally to B, it is possible to have smooth R and standard N, while externally under wellfoundedness this cannot happen.

Thus, we arrive at the well-founded Boolean ultrapower ${\overset{ˇ}{V}}_{U}$. Then, it holds that

**Lemma** **8** ([15], Lemma 24)**.** *If ${\overset{ˇ}{V}}_{U}$ is wellfounded, then so is $V^{B}/U$. In this case, the Mostowski collapse of $V^{B}/U$ is the forcing extension $\bar{V}\left[G\right]$ of the transitive model $\bar{V}$ of ZFC arising as the collapse of ${\overset{ˇ}{V}}_{U}$.*

Given *UV*-generic and wellfounded ${\overset{ˇ}{V}}_{U}$, we have standard transitive $\bar{V}$ and its (standard transitive) random forcing extension $\bar{V}\left[G\right]$. Thus, by starting with nonwellfounded $\hat{U}$ in *B* (which always exists according to Lemma 6) and requiring the standardness of N, we end with random forcing extension of reals. This shows the correctness of Lemma 5.

Now, let us turn to Theorem 1. In Figure 2, the filled arrow 1 shows how manifold-like $R^{4}$ is represented in L—i.e., s(R) is just the smooth ring $C^{\infty }\left(R\right)$ in L and $s\left(R^{4}\right)=C^{\infty }\left(R\right)\times_{\infty }C^{\infty }\left(R\right)\times_{\infty }C^{\infty }\left(R\right)\times_{\infty }C^{\infty }\left(R\right)$. Here, $\times_{\infty }$ is the coproduct in L. This L is then Yoneda embedded into ${\mathrm{Set}}^{L^{op}}$, which leads to its representability in B (see Remark 6). This is the arrow 2. The existence of nonstandard numbers in B (Remark 6) gives the connection with the nonstandard numbers in a 2-valued, external, context. To complete the proof, let us recall what we require from a smoothness structure on $R^{4}$ to agree with QM-generated smoothness. On the QM side, there have to be local patches in the smooth cover of $R^{4}$, which, after formalization, live in the random forcing extensions of a model *M* of ZFC. Moreover, there has to be more than one such local patch in different forcing extensions [6,12]. On the smooth B-spacetime side, we have local patches on a smooth cover of $R^{4}$, where some of them live in B as isomorphic copies of the internal object $R^{4}$ (*R* is the object of smooth real numbers). Both 2-valued and smooth limits of the QM and B-spacetime sides meet in a smooth manifold, which formally should be described in a wellfounded and 2-valued formal model. We call this resulting smooth manifold a B-invariant set-based smooth manifold. The Lemma 5 now shows that the two ends, quantum and B-smooth, meet consistently relative to local patches on a smooth cover of $R^{4}$. The arrow 4 indicates the forcing extension coming from the Mostowski collapses of ${\overset{ˇ}{V}}_{U}$ and $V_{U}^{B}$, as in Lemma 8. Thus, Theorem 1 holds true.

The resulting smoothness agreeing with the both ends has to be some exotic $R^{4}$. The assignment of some exotic $R^{4}$ in the B-smooth case represents Lemma 1 [8], and for QM it was shown in [12]. The present result shows that they agree on the formal level and that the smoothness can be seen as a driving force of the evolution of the universe (e.g., [3]) and an important formal feature of the future reconciliation of QM and GR. We will see in the next section how the existence of such smoothness can modify our understanding of the information loss paradox connected with black holes.

Whenever BH is formed in spacetime, this process also affects spacetime itself. This is the modification of the smoothness structure on 4-dimensional regions, which eventually propagates and extends over cosmological big regions. We found that this modification can be represented as a change in the smoothness structure, such that the modified smoothness on $R^{4}$ should also match the quantum regime and, as such, should be locally driven by B. The process from the formal point of view does not distinguish this or other smoothing $R^{4}$s, but rather is broad enough to capture small, large, or even alleged smoothness underlying would-be exotic 4-spheres. At this stage, we cannot indicate precise smoothing emerging or negate the possibility that various smoothings appear depending on the physical processes connected with the forming or evaporation of BHs. Once the structure of smoothness on spacetime 4-regions is modified, it cannot be erased by any diffeomorphism transformation of the regions (there is no diffeomorphism to the standard $R^{4}$).

## 4. Information Loss Paradox

The results obtained so far show that the existence of BHs in spacetime—i.e., with singularities extending them—indicates a smoothness structure locally modified by B. The large-scale smoothness of the 4-regions of spacetime is thus exotic. This scenario raises several questions and leads to new possibilities. One such possibility involves the fate of information seemingly lost when BH evaporates (e.g., [24,25]). The formalism in the above sections shows that if there is a BH in spacetime, the smoothness is modified so after evaporation this can be a carrier of information involved in the process of BH formation. After evaporation, we are not left with just spacetime without BH (with thermal radiation spreading out the spacetime), but the smooth structure of spacetime is radically changed over large scales (even though locally it remains the same); see the following lemma [12] (Corollary 1).

**Lemma** **9.** *The QM lattice of projections L is the source of the non-vanishing large-scale curvature on $R^{4}$. This curvature cannot be removed by any coordinate diffeomorphism of $R^{4}$.*

This process of changing smoothness is not described within the standard GR, where one instead fixes smoothness on the spacetime manifold and then solves Einstein’s equations. The change in smoothness can, however, contain quantum information not only about matter fields in spacetime but also about gravity in the quantum regime. An indication comes from QG, where in the path integral one sums over all possible smoothings of spacetime. To understand this smoothings/quantum connection more clearly, let us consider the largest member in the Gompf–Taubes radial family of large $R^{4}$s [7,14]. This $R^{4}$ is a Riemannian Ricci flat but also hyper-Kähler [7] (Corollaries 1.2, 1.3) and thus a gravitational instanton. The change in the smoothness is thus represented by a ’tunnelling’ to a gravitational instanton which probably requires some QG treatment. Assuming that the large-scale curvature of Lemma 9 comes from the large $R^{4}$ shows that the evaporating BH could leave a quite nontrivial state of spacetime, the description of which refers to unknown QG processes. Pushing this point of view further, there is the issue of causality violation, which is connected implicitly with the appearance of large exotic $R^{4}$ within spacetime. Namely, this $R^{4}$ breaks the strong cosmic censorship conjecture in a sense that $R^{4}$ allows for a Lorentzian Ricci flat (possibly incomplete) smooth metric, and there exist an abundance of such 4-manifolds; thus, the situation of breaking is generic in dimension 4 [7] (Theorem 1.3). Each of such $R^{4}$ is homeomorphic to $R^{4}$, although they cannot be represented as a globally hyperbolic smooth Lorentzian manifold (as with any smooth global product $R\times M^{3}$ for any $M^{3}$). This shows that, at large scales, the hyperbolicity breaks, even though locally this is undetected by any observational means. We think this phenomenon will find its place as a valid contribution to the final theory of QG. One might see that the nonvanishing curvature of $R^{4}$, which cannot be removed by any diffeomorphism, (globally) reflects a fundamental property of spacetime and sheds new light on the quantum/classical link of gravity and spacetime. Another important indication comes from the approach called *smooth* QG, where smoothness is not only deeply linked with QG but can rather generate some quantumness of gravity based on the highly nontrivial topology and geometry of 3- and 4-dimensional submanifolds involved in the process of describing the rich structure of 4-smoothness (e.g., [5]). Exotic smoothness of this kind would be indestructible in 4-spacetime even in the deepest quantum limit, notwithstanding that we do not follow such radical possibility here.

Another component of storing information in the smoothness structure of spacetime, which has been modified after the evaporation of BHs, is quantum matter. We still do not have a full microscopic description of the process; however, important theoretical facts have been collected from several years of studies attempting to clarify this issue. Again, we focus on the exotic smoothness of 4-dimensional regions of spacetime which might have been modified during the BH evaporation. From a general standpoint from the basis of differential geometry and topology, one finds ways in which fermionic and bosonic quantum fields are representable in the smoothness structures on $R^{4}$. There are several results relating to matter fields and exotic 4-smoothness (e.g., [26,27,28,29]). One might think that exotic $R^{4}$s as topological manifolds are trivial, since they are all homeomorphic to $R^{4}$. However, the 3-dimensional structure of their 3-submanifolds is unusually complicated and we are still far from understanding it. This situation is more tractable for small exotic $R^{4}$s, where we understand their handlebody structures comparatively well [14].

**Remark** **7.** *Given an exotic $R^{4}$, let us remove from it a 4-disk. Then, topologically, the result is an open 4-manifold $R^{4}\backslash D^{4}=S^{3}\times R$. However, the manifold $S^{3}\times R$, although topologically trivial, cannot be diffeomorphic to its standard smoothness structure generated by the smooth global product. In fact, $S^{3}\times R$ carries infinitely many nondiffeomorphic smoothness structures which are called exotic $S^{3}\times R$ [14]. Among the exotic smoothings of $S^{3}\times R$, there are ones discovered and described by Freedman (fake $S^{3}\times_{\theta }R$) which do not come from any exotic $R^{4}$, as shown above [14].*

Taking exotic $S^{3}\times R$ as a model for the expanding universe, one can determine several observed parameters. For the standard $S^{3}\times R$, such derivation is not possible, since certain nontrivial topology changes between the 3-dimensional submanifolds of $S^{3}\times R$ do not occur (become trivially $S^{3}\to S^{3}$) [3,29,30]. Thus, allowing for exotic $R^{4}$ and exotic $S^{3}\times R$ in the model of the universe, one can purely topologically determine the realistic GUT and electroweak scales of energies and masses, which are then used in the seesaw mechanism of the particle physics, leading to the reliable values for the neutrino masses [29]. In [28], the three different incompatible smoothings were considered—namely, exotic $S^{3}\times_{\theta }R$, exotic $R^{4}$s, and the standard R. It was observed that their incompatibility as smooth 4-manifolds might be responsible for certain problems with the final formulation of QG. Moreover, the Higgs boson mass is realistically encoded in the specific exotic smoothness $S^{3}\times_{\Sigma (8_{10})}R$ determined by the homology 3-sphere $\Theta =\Sigma (8_{10})$ [28]. Thus, the general formalism of [26,27], showing how exotic $R^{4}$s carry information about fermionic or bosonic fields, results in the realistic topological encoding of certain fields of the standard model of particles.

Moreover, as we noted already above, applying exotic smoothness in building models of the universe gives rise to important results, such as determining (topologically) the realistic value of the cosmological constant [3] or certain cosmological parameters such as the number of *e*-folds during inflation or the α parameter in the Starobinsky model. The nontrivial intersection of exotic smoothness in dimension 4 with the formalism of QM augmented by the topological support of various fundamental physical quantities clearly indicates that the exotic 4-smoothness of spacetime uncovers certain fundamental layers of spacetime and gravity. This, more than any exotic $R^{4}$, cannot be a flat Riemannian 4-manifold, meaning that there is a kind of intrinsic ’topological’ gravity assigned to exotic smooth spacetimes. The BH remnant in spacetime, if gathered in an exotic smoothness structure, is still nontrivial and indicates the underlying quantumness of the process. BHs evaporate, leaving spacetime with a modified smoothness which still contains information about quantum matter and fields. Moreover, the structure resulting from a kind of primordial BH can be used to develop a realistic, topologically supported, cosmological scenario for our universe.

The precise physical meaning of the above observations remains to be clarified, as does a better understanding of the quantum regime of gravity. Here, we show that spacetime itself, before eventually completing quantum dismounting, encodes nontrivial (grasped here by formal means) information regarding cosmological and quantum microscopic scales. This encoding is, however, intricate, and uncovering a clearer message requires additional effort by theoreticians; however, we believe this will be available for experimental scrutiny.

## 5. Discussion

In this work, we applied the method of local formalization in spacetime. This method leads to recovering the formal contexts of ZFC models assigned to different regions in spacetime. As the result, we found well-defined discrepancies between the formal structures in different regions. When BH is formed in spacetime (by a matter/energy collapse), this method can be extended such that the descrepancies of the singular quantum region and smooth cosmological 4-domain are expressible in terms of random forcings and B-local modifications of spacetime. We show in Theorem 1 that there is well-defined smoothing on $R^{4}$, which agrees with both scales in spacetime: the micro and cosmological scale. We reinterpreted this finding in such a way that the smoothness of spacetime at large scales is modified so that it can carry quantum information from the quantum BH singularities. This particular feature can also be understood as the very basic property of exotic smoothness—i.e., it cannot be reduced to an atlas which contains a single local patch; otherwise, the structure collapses to the standard one. In quantum terms, this means that quantum entanglement in pure states is translated into the irreducible multiplicity of local patches on 4-dimensional regions of spacetime; otherwise, the entanglement would become statistically reduced to a mixture of states. The property seen at the quantum lattice L side means that it cannot be collapsed, or reduced, to a single Boolean algebra; thus, it has to allow for entangled quantum states. If an exotic smoothness is the geometric remnant of evaporating BH, it still can store the quantum information about entanglement and pure states. In this sense, the spacetime structure by itself encodes quantum states of BH as their remnants. We should stress that even though the presented approach looks promising, the goal of finding a definite solution to the information loss paradox would require direct ’microscopic’ calculations showing precisely how the emergence of exotic smoothness from the quantum regime actually occurs. On the one hand, this should be accessible for the future full QG theory. However, on the other hand, the effort in this paper parallels the effort of searching for a successful QG. Similarly, the issue of noncausality also needs to be addressed carefully in the scope of QG, though the way presented here—i.e., via exotic 4-smoothness [7]—can be a valid contribution to the final understanding of this problem. Moreover, it is currently undecided which smoothing emerges as the remnant after the specific BH evaporation. Even for exotic $S^{3}\times R$, we have those of Freedman or coming from exotic $R^{4}$s, and it seems that both can have a physical meaning. A full QG should answer such questions.

Independently of the questions related to BHs, a nontrivial spacetime structure has been proposed where various exotic smoothings $R^{4}$s correspond to excited states of the spacetime itself. Spacetime thus gains canonical ’quantum-like’ states, which gives the way to considering it as a quantum system. Such a nontrivial pattern has been used in the attempt to explain the vanishing contributions to the density of energy coming from various quantum fields in spacetime [6]. The complementary problem—i.e., how to obtain the extremely tiny realistic value of the cosmological constant—also found its solution in the realm of exotic 4-smoothness [3]. Returning to the early ideas around so-called Brans conjecture, stating from the fact that exotic smoothness in spacetime can act as a nontrivial gravitational external matter source, the approach presented here can be seen as a quantum generalization.

## Data Availability

Not applicable.

## Abbreviations

The following abbreviations are used in this manuscript:

ZFC	Zermelo–Fraenkel set theory with the axiom of choice.
B; B	the smooth Basel topos; the site for B.
*B*	the atomless random Boolean algebra.
N; ${}^{*}N$; N	the object of smooth N.N; nonstandard N.N.; the standard set of N.N.
Set	the category of sets and mappings.

## Appendix A. Schwarzschild BH Singularity and Exotic Smoothness of 4-Spacetime

We give examples (following [12]) showing that the presence of quantum singularities connected with Schwarzschild BH in 4-spacetime gives rise to the modified smoothness of the spacetime, which can be considered as the remnant of evaporating BH. We work with Schwarzschild BH to contain spacetime singularity, which has to extend the classical description of spacetime. The proper understanding of this essentially requires QM. The consistent complete description of the singularity requires a theory of quantum gravity, for which a final formulation is still missing. However, the form of energy and matter due to this QG regime follows the QM rules; thus, we assume two limiting but complementary regimes, one with smooth spacetime containing the event horizon, the other being connected with the singularity, represented by a QM Hilbert space H with the lattice of projections L(H). The singularity of the Schwarzschild BH is Weyl-type, since the Weyl tensor diverges [31] (p. 146). The large-scale smoothness structure of the spacetime containing the Schwarzschild BH solution is thus modified by the presence of the singalarity, which essentially requires QM description.

The Schwarzschild metric within GR around a black hole in the Kruskal presentation reads as

$$ds^{2}=\frac{2M^{3}e^{-r/2M}}{r}\left(-du^{2}+dv^{2}\right)+r^{2}d\Omega^{2}$$

where $d\Omega^{2}=d\theta^{2}+\sin^{2}\theta d\phi^{2}$ is the standard spherical metric on $S^{2}$, and u,v are coordinates on $R^{2}$, such that $u^{2}-v^{2}=\left(\frac{r}{2M}-1\right)\left(e^{r/2M}\right)$. The Krtuskal coordinates u,v,θ,ϕ thus parameterize the manifold $R^{2}\times S^{2}$ by $u^{2}-v^{2}\leq 1,\left(\theta ,\phi \right)\in S^{2}$.

Let us now see how the smooth geometry of this BH is forced to be exotic due to the QM lattice L(H). The manifold $R^{2}\times S^{2}$ is a smooth submanifold of $R^{4}$, since

$$R^{2}\times S^{2}=R^{4}\backslash D^{3}\times R$$

where $D^{3}$ is the closed 3-disk in $R^{4}$. Thus, geometrically, the region containing the Schwarzschild singularity of BH is now placed in the complement of $R^{2}\times S^{2}$—i.e., in $D^{3}\times R$. Now, Theorem 3 in [12] states that $R^{4}$ cannot be standard smooth and is thus exotic smooth $R^{4}$. From the perspective of the energy/matter leading to the creation of BH in the singular region above it, the quantum description of H, with L being the lattice of projections. Let ba(L) be the set of all complete maximal Boolean algebras of projections from L. Theorem 3 of [12] ensures that the sources $T_{\mu \nu }$ modify the large-scale smoothness structure of $R^{4}$, such that it cannot be diffeomorphic to the initial standard smoothness structure, which means that ba(L) cannot be reduced to a single maximal algebra. However, the original smooth Kruskal metric on $R^{2}\times S^{2}$ undergoes an important modification too: it becomes smooth but is not a globally smooth product $R^{2}\times S^{2}$, merely being a topological product. The resulting smooth geometry $R^{2}\times_{T_{\mu \nu }}S^{2}$ is not diffeomorphic to the standard product, as it was analyzed previously and is known as *exotic* Schwarzschild BH [4,32]. We can state briefly that the exotic smoothness of $R^{4}$ enforces the exotic smoothness of the Kruskal geometry $R^{2}\times S^{2}$. Thus, in GR energy–momentum sources modify the curvature of spacetime and their quantum nature leads to the modification of the smoothness structure. These two effects together can help us to understand the explanation of the information loss paradox as in this paper: the prolongation backward in time of the smooth standard Kruskal coordinates meets obstructions (non-global product) which can be interpreted as additional energy–momentum sources. The evaporating BH encodes the sources in its remnant, which is the exotic smoothness of spacetime agreeing with the exotic Kruskal geometry. No 4-diffeomorphism of such a region can diminish the exotic curvature; thus, within GR one cannot erase this effect and the remnant is stable from that point of view.

**Remark** **A1.**
*Note that the entire approach works exclusively in dimension 4: only for n=4 does there exist an exotic $R^{n}$ as well exotic $S^{k}\times_{T_{\mu \nu }}R^{l}$ for k+l=4, k,l≥1,k,l∈N. In particular, exotic Schwarzschild BH are entirely possible in dim. 4 for k=l=2.*

We would be very interested in approaching the modified exotic metric analytically and showing through calculations how it emerges in a true physical process. However, at present nobody knows any analytic presentation of exotic metric on any $R^{4}$; thus, we are left with general topological and geometric (or even categorical) considerations, as discussed in the paper. Possibly, a solution could be to perform certain numerical chasing for exotic metrics in the spirit of, e.g., [33,34]. However, right now this requires further careful analysis as well as insights (maybe mainly) based on theoretical grounds.
